# Quantitative account of social interactions in a mental health care ecosystem: cooperation, trust and collective action

**DOI:** 10.1038/s41598-018-21900-1

**Published:** 2018-02-28

**Authors:** Anna Cigarini, Julián Vicens, Jordi Duch, Angel Sánchez, Josep Perelló

**Affiliations:** 1 Departament de Física de la Matèria Condensada, Universitat de Barcelona, 08028 Barcelona, Spain; 2 Universitat de Barcelona Institute of Complex Systems UBICS, 08028 Barcelona, Spain; 3 Departament d’Enginyeria Informàtica i Matemàtiques, Universitat Rovira i Virgili, 43007 Tarragona, Spain; 4 Northwestern Institute on Complex Systems (NICO), Northwestern University, 60208 Evanston, IL USA; 5 Grupo Interdisciplinar de Sistemas Complejos (GISC), Unidad de Matemática, Modelización y Ciencia Computacional, Universidad Carlos III de Madrid, 28911 Leganés, Spain; 6 Unidad Mixta Interdisciplinar de Comportamiento y Complejidad Social (UMICCS) UC3M-UV-UZ, Universidad Carlos III de Madrid, 28911 Leganés, Spain; 7 Institute UC3M-BS of Financial Big Data, Universidad Carlos III de Madrid, 28903 Getafe, Spain; 8 Instituto de Biocomputación y Física de Sistemas Complejos (BIFI), Universidad de Zaragoza, 50009 Zaragoza, Spain

## Abstract

Mental disorders have an enormous impact in our society, both in personal terms and in the economic costs associated with their treatment. In order to scale up services and bring down costs, administrations are starting to promote social interactions as key to care provision. We analyze quantitatively the importance of communities for effective mental health care, considering all community members involved. By means of citizen science practices, we have designed a suite of games that allow to probe into different behavioral traits of the role groups of the ecosystem. The evidence reinforces the idea of community social capital, with caregivers and professionals playing a leading role. Yet, the cost of collective action is mainly supported by individuals with a mental condition - which unveils their vulnerability. The results are in general agreement with previous findings but, since we broaden the perspective of previous studies, we are also able to find marked differences in the social behavior of certain groups of mental disorders. We finally point to the conditions under which cooperation among members of the ecosystem is better sustained, suggesting how virtuous cycles of inclusion and participation can be promoted in a ‘care in the community’ framework.

## Introduction

Approximately one fifth of the world population will suffer some mental disorder (MD) at some point in their lives, such as anxiety or depression^1^. The direct economic costs of MD, including care and indirect effects, is estimated to reach $6 trillion in 2030, which is more than cancer, diabetes, and respiratory diseases combined^2^. As part of a global effort to scale up services and bring down costs, reliance is increasingly made upon informal social networks^3^. A holistic approach to mental health promotion and care provision is then necessary, and emphasis is placed on the idea of individuals-in-community: individuals with MD are defined not just alone but in relationship to others^4^. Such a paradigm shift implies superseding the traditional physician-patient dyad to include caregivers, relatives, social workers, and the community as a whole, recognizing their crucial role in the recovery process.

A key aspect in the definition and aetiology of MD has to do with social behavior^5^: behavioral symptoms, or consequences at the behavioral level, characterize most MD. For instance, autism, social phobia, or personality disorders are determined by the presence of impairments in social interaction. Other disorders result in significant difficulties in the social domain, such as depression or psychotic disorders. Further, conditions that are intrinsically behavioral (as for eating disorders or substance abuse) seem to be exacerbated by the influence of social peers. A large body of research has therefore looked at the neural basis of social decision-making among individuals with MD to identify objective biomarkers that may prove useful for its diagnosis, therapy evaluation, and understanding^6–8^. However, such a methodology does not well fit into the individuals-in-community paradigm. We argue that an agent-based approach which draws upon experimental game theory might prove insightful and ecologically valid for the study of behavior in a given social environment.

Within the mental health literature, the use of game theory as a way to understand the multi-faceted dimensions of behavior has received already quite some attention^9,10^. Most research addressed the issue of behavioral differences between individuals with MD and healthy populations^6,7,11–16^. These works, that point to cognitive and affective processing impairments^6,16,17^, further support the idea that MDs are associated with significant and pervasive difficulties in social cognition and altered decision-making at various levels. Yet, despite these studies are of very much interest, they are primarly concerned with dyadic interactions among people with specific MDs. That is, they lack insights into the complexity of individual behaviors of MD within a specific social context.

Here we adopt a novel community perspective. Our objective is twofold: First, we aim to develop a thorough taxonomy of the behavioral traits of role groups within the collective. We thus account for both the heterogeneity of actors, and for multiple types of social interactions. We strongly believe that to predict and understand behavior is necessary to consider the relationship context in which individuals are embedded. Therefore diversity of roles, motivations or capabilities, must be taken into account. Also, real life social interactions occur in different forms; sometimes people must work together, some others they have to coordinate or anti-coordinate their behaviors, yet in other situations they find themselves in more or less disadvantaged positions. It is therefore of crucial importance to encompass a comprehensive range of strategic situations if we are to appreciate behavior. That is, traits such as trust, altruism, or reciprocity, along with the person’s own expectations, all play a role in the process of decision making in social contexts. This calls for an experimental approach in which participants face several strategic settings. Our second objective is to provide quantitative accounts of social capital within the mental health community, bringing the notion of social capital into the forefront of mental health care. Far from being universally defined, its core contention is that social networks are a valuable asset, providing a basis for social cohesion and cooperation towards a common goal^18^ (which is, in our case, mental care provision). It thus encompasses those norms and forces that shape social interactions, serving as the glue that holds society together^19^.

For these purposes, we have designed an experimental setup that probes into the complexity of the interdependencies at play within the mental health ecosystem. Accordingly, our experiments take place in a socialized, lab-in-the-field setting^20^, in order to be as close as possible to the dynamic and unique nature of real-life social interactions. The design of our socialized setup is based on a participatory process and citizen science practices^20^ which counted on the collaboration of all stakeholders of the mental health ecosystem. By combining all these ingredients, we have developed a framework that, as will be shown below, allows to capture some difficult-to-observe aspects of behavior and social capital within mental health ecosystems as a way to understand how communities contribute to care and resocialization.

## Results

A full description of the games we implemented can be found in the Methods section below, but for clarity we briefly describe here the games we used. We had participants play two dyadic games, namely the Trust game, in which they had to lend money to another player who then obtains a return, and has the option to send some money back to the lender; players played in both roles. They also played the well known Prisoner’s Dilemma, in which they had to choose to cooperate or to try to benefit from the other’s cooperation. Finally, they played a collective risk dilemma, in which the whole group had to reach a common goal to avert a catastrophe that most likely would wipe out their money. Participants belonging to the mental health ecosystem played with each other in group of six players. However, they could by no means guess with whom they were actually playing.

We begin the presentation of our results from the dyadic games of our suite of strategic interactions. Aggregate behavioral measures point to systematic deviations from self-interested predictions which are in line with previous literature on experimental game play^21^. In the Prisoner’s Dilemma (PD), the average cooperation rate across all individuals is *c* = 0.61 ± 0.03 (standard error of the mean), which is notoriously well above the Nash equilibrium prediction of *c* = 0. Participants behavior in the PD is also significantly associated with their estimates about the likely cooperation of the partner (${\chi }^{2}=32.48$, *p* = 1.2 · 10^8^), with 44% of all participants expecting the partner to cooperate, and thus cooperating themselves. This points to the crucial role of positive expectations on cooperative behavior^22^. Further, participants trust and reciprocate positive amounts in the Trust Game (5.79 ± 0.15 monetary units (MU) and 41.3 ± 1.37% of the amount available to return, respectively), again departing largely from Nash equilibrium conjectures of 0 MU transferred. The results also suggest that in considering the mental health community in its whole, thus accounting for the diversity of actors and roles, the global picture does not substantially differ from society at large.

### Sectorial and dyadic behavior

**Figure 1:**
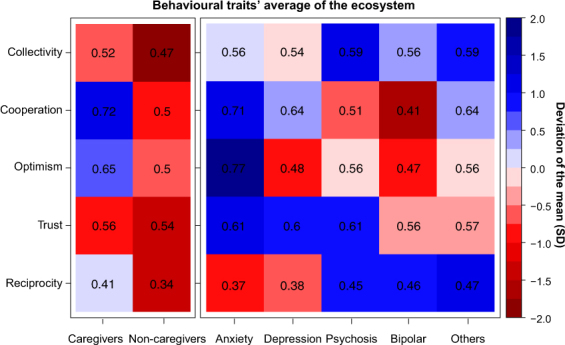
Heatmap of behavioural traits’ average and deviation of the mean across games. Collectivity refers to the ratio of contribution in the Collective-Risk Social Dilemma. Cooperation and Optimism refers to the ratio of cooperation and expected cooperation, respectively, in Prisoner’s Dilemma. Trust and Reciprocity refers to the ratio of capital trusted and reciprocated in Trust Game. The left part shows the ratio of individuals without mental conditions: caregivers (professionals and relatives with caregiving tasks) and non-caregivers (relatives without caregiving tasks, friends and others). The right part shows the actions of individuals with mental conditions. Therefore, the number in each cell indicates the ratio of social preferences per subjects in each social dilemma and the color scale shows the deviation of the mean measured in SD units.

As we stated above, our main interest is to delve into the behavior of the different actors who make up the mental health ecosystem Fig. 1 summarizes the results for the five groups of individuals concerned. The heatmap yields several insights that are worth commenting upon.

In one-shot dyadic interactions some marked differences in the frequency of cooperative behaviors (PD) arise within the collective formed by affected with MD, caregivers, non-caregivers (Kruskal-Wallis rank sum test, $H=6.04,df=2,p=0.0488$). Further pairwise comparisons (see Supplementary Table S1) show that participants with anxiety and caregivers are more likely to opt for the cooperative strategy compared to participants with bipolar disorder, psychosis or other members of the collective. Participants with anxiety are also the ones with the most positive expectations about the partner’s behavior compared to all but caregivers (see Supplementary Table S2). Also, relatives, friends and other members with no MD defect more than caregivers (Mann-Whitney U test, $U=1352,p=0.02839$), being relatives remarkably less cooperative than the rest of the collective *c* = 0.33 ± 0.16. This suggests that cooperation among members of the mental health ecosystem is contextually based, depending on the role that actors play in the recovery process. It also varies across diagnostics, revealing a marked cooperativeness and optimism of individuals with anxiety disorders.

On the other hand, in sequential dyadic interactions (TG) all participants trust more than half of their endowment, being the distribution of initial transfers similar across groups. No variation is indeed found in trust levels between participants with MD, caregivers and non caregivers (Kruskal-Wallis rank sum test, $H=2.75,df=2,p=0.25$). Yet, at the time of reciprocating the partner’s behavior, participants with anxiety and depression return the least (37.5 ± 3.3%). The difference is significant if compared to return transfers of participants with psychosis or other diagnostics (see Supplementary Table S4).

### Group interaction

Our experimental setup has proven extremely informative in its most novel section, namely the analysis of group interactions framed within the Collective Risk Dilemma (CRD), with no prior result within the mental health literature. In global terms, the average amount contributed to the public good (22.6 MUs) is much more than the fair contribution of 20 MUs, where by fair we understand sharing equally the total amount needed for the threshold (120 MUs) among all six participants. Here it is important to keep in mind that participants were told that all money contributed would go to reforestation projects, so it is not irrational to keep contributing beyond the threshold as many of our subjects did. The key result in the CRD is that large, significant differences (t-test, $t=2.85,df=242,p=0.0047$) are found between participants with and without mental disorders. The former contribute with 22.95 ± 0.63 MUs compared to 20.34 ± 0.68 MUs from the latter, and therefore it appears that when repeated interaction and sustained teamwork (CRD) are required, people with MD contribute much more to the common goal (See Supplementary Section 1.6.2).

**Figure 2:**
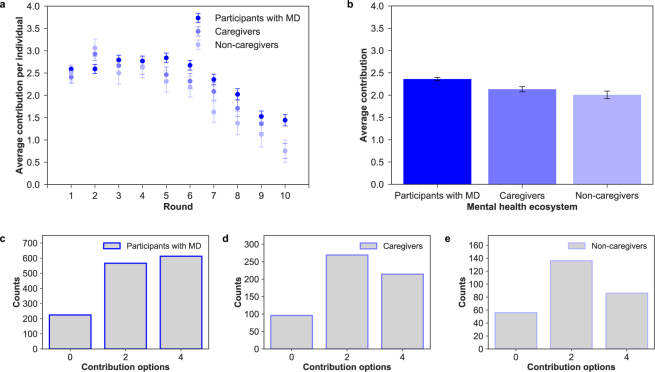
**(a)** Individual contribution over rounds. Evolution of contributions (mean and standard error of the mean) during the game between participants with mental disorder conditions, caregivers and non-caregivers. We can see that all groups behave similarly and in an identical way to a previous experiment run outside the mental health ecosystem^40^. **(b)** Average individual contribution per round. Average contribution and standard error of the mean in the mental health ecosystem. There are significant differences between participant with MD and the rest of actors, caregivers (t-test, $t=2.107,df=155,p < 0.0294$) and non-caregivers (t-test, $t=2.499,df=48,p=0.01588$). Distribution of choices by participants with MD (**c**), caregivers **(d)** and non-caregivers (**e**). The most of participants with MD (43.6%) selected the maximum contribution (4), while the caregivers (46.5%) and non-caregivers (48.9%) mostly selected the fair contribution (2).

Contribution dynamics vary according to group composition in terms of number of participants with mental disorder conditions and other actors involved in the recovery process. All groups successfully reach the target collecting on average 135.64 ± 1.75 MUs (see Supplementary Section 1.6.1). Similarly to other public good experiments, contributions decrease over time^23^. While in the first round participants contribute around 56.3% of the allowed contribution per round (2.2 ± 0.07 MUs, where the social optimum is 2 MU), contributions drop when the endgame effect sets in. A Spearman’s rank-order correlation of contributions over rounds corroborates this negative time trend ($\rho =-0.757,p < 0.05$). Both patients and actors involved in the recovery process reduce their contributions by the end of the game. However, in almost all rounds, participants with a mental condition contribute more than caregivers and non caregivers, for whom motivations to contribute decline steadily (see Fig. 2).

**Figure 3:**
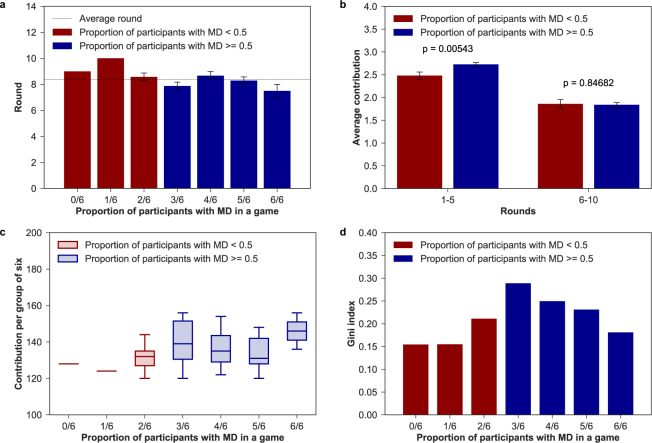
**(a)** Average round of achievement. Round (mean and standard error of the mean) in which the group of six achieved the target. **(b)** Aggregated contributions per group composition. Contributions (mean and standard error of the mean) in the first and last five-rounds per number of individuals with MD in a group. There are significant differences (t-test *p* < 0.01) in contributions in the first part of the game. **(c**) Contributions per group of six. Total group contributions by number of individuals with mental conditions in the group. **(d**) Gini index of final payoff within groups. Level of inequality in final payoff based on the number of individuals with MD in each group.

In terms of the group composition, groups where individuals with MD conditions constitute half or the majority of the group (n = 36) do much better in sustaining cooperation compared to groups where firsthand affected are the minority (n = 9). It is here worth to mention that participants may see who the rest of the members are but ignore who is exactly making the choice in the game (see Methods for further details). As Fig. 3b shows, while average individual contributions are similar in the last periods (rounds 6–10 t-test, *t* = 0.19, *p* = 0.85), groups with half or more individuals with MD contribute significantly more at the beginning of the game (rounds 1–5 t-test, *t* = 2.79, *p* = 0.0054). Hence, the presence of three or more individuals with a mental condition in the group has a positive and stabilizing effect on average individual contributions. Likewise, in games with a low proportion of participants affected with MD the group achieved the goal, on average, later than in games with more than 50% of participants affected with MD (see Fig. 3a).

If we then break down the analysis by group type, we find that group members contribute and benefit differently from cooperation (see Fig. 3c). Indeed, final payoffs within groups are far from being equally distributed (see Fig. 3d), with the highest inequality found in the group where the number of patients equals the number of actors involved in the recovery process (Gini coefficient = 0.289). We thus see clearly that the cost of collective action is mainly supported by individuals with a mental disorder. Given that they contribute the most within all groups, lower investments are needed for other members of the collective to reach the common target. Yet, in 4/6 and 5/6 groups caregivers reduce average individual contributions while non-caregivers pay more than their fair share. In 1/6 and 2/6 groups, on the other hand, caregivers are the ones who compensate the unfair contributions of other members. These last groups are the ones that ensure the lowest inequality in final payoffs. Therefore, while our results are unambiguous about the larger readiness for collective action among people with MD, we cannot claim nothing about the rest of the collective.

## Discussion

Let us now turn to the discussion of the above results and their implications (see Table 1 for a summary of the key findings). As a first general remark, through our lab-in-the field experiment we found that an ecosystem approach to mental health care brings with it a quite complex scenario with several interesting insights. To begin with, participants with anxiety symptoms display a markedly different behavior compared to other diagnostics: they are more likely to opt for the cooperative strategy compared to individuals with bipolar disorder or depression, and return significantly less than participants with psychosis or other disorders. Since the current study is the first to investigate social decision-making within a heterogeneous population of individuals diagnosed with MD, a comparison with previous research is only possible referring to studies focusing on specific clinical and quite homogeneous populations. Several experiments have demonstrated deficits in cooperative behavior among individuals with anxiety or depression when playing iterated versions of the PD^11,17,24,25^, but results about altruism (Ultimatum Game) and trust are inconsistent between studies^6,7,11,12,17,26^. Individuals with major depressive disorders (which include anxiety and depressive symptoms) have also been found to systematically differ when their emotional responses to fairness are compared^6,17^, showing higher levels of negative feelings when faced with unfair treatments. One of the hypothesis advanced to explain the systematic behavioral differences of individuals with anxiety relates to a potentiated sensitivity to negative stimuli as well as a tendency to treat neutral or ambiguous stimuli as negative or as less positive^6,12,17,27^. This hypothesis might find support in our results as for the low returns in the Trust Game, despite displaying relatively high trust in the partner’s behavior and very high expectations. Indeed, participants with depressive or anxiety symptoms in our experiment significantly over-punish trustee transfers, but the low returns are independent of the amount received. This seems to imply that participants with mood disorders respond negatively to their partner behavior, as if they interpret their partner’s choice in a negative sense. Alternatively, fairness considerations may be playing a role: low returns of participants with mood disorders might therefore be due to different fairness perceptions^6,12,17^, which result in a bias towards negative reactions rather than positive rewarding.

**Table 1:** Summary of the key findings from the suite of dyadic and repeated interactions among members of the mental health ecosystem.

**Participants with anxiety display markedly different behavior compared to other diagnostics**.	
• More likely to opt for the cooperative strategy compared to participants with bipolar disorder.	MWU test *p* < 0.05.
• Associated with the most positive expectations about the partner’s behavior.	*c*_*exp*_ = 0.77 ± 0.08. MWU tests comparisons across diagnostics *p* < 0.05.
• Show a significantly high frequency of cooperative interactions compared to individuals with bipolar disorder or depression.	*c* = 0.71 ± 0.08 MWU tests comparisons *p* < 0.05 and *p* < 0.1.
• Return significantly less than participants with psychosis or other disorders.	*r* = 0.37 ± 0.05. MWU test comparisons *p* < 0.1.
**Cooperation depends on the role that actors play in the recovery process**.	
• Significant differences in the frequency of cooperative interactions across role groups.	KW-RS test, *p* < 0.05.
• Caregivers contribute with large degrees of cooperativeness and optimism.	*c* = 0.72 ± 0.05, *c*_*exp*_ = 0.65 ± 0.06.
• Relatives are the weak links of the ecosystem.	*c* = 0.33 ± 0.16, *c*_*exp*_ = 0.44 ± 0.18.
**Individuals with MD support the cost of collective action**.	
• MD contribute more than caregivers and non caregivers to the public good.	Independent t-test *p* < 0.005. Average contributions: 22.95 ± 0.63 MUs, and 20.34 ± 0.68 MUs respectively.
• Groups with half or more MD do better in sustaining cooperation in the first rounds.	Independent t-test *p* < 0.01.
• Inequality in the distribution of final payoffs is at his maximum in groups where MD constitute half of the group.	Gini coefficient 0.289.

MWU: Mann-Whitney-U, KW-RS: Kruskal-Wallis rank sum, *c*: cooperation level, *c*_*exp*_: expected cooperation level, *r*: return ratio in Trust Game, and MUs: monetary units.

Deficits in economic game play have also been documented for individuals with bipolar disorder. Studies report low and decreasing trust levels over sequential interactions, skeptical beliefs about the partner’s behavior and a tendency to break cooperative interactions^28,29^. Again, this is partly supported by our results. Negative expectations of participants with bipolar disorder indeed agree with a low frequency of cooperative choices, little amounts of money sent to trustees, and low contributions to collective action. In line with King-Casas *et al*. results^29^, while individuals with depression trust in the cooperativeness of other people, those with bipolar personality disorders do not. Cognitive dysfunctions (insula response) might possibly reflect an atypical social norm in this group^29^. Consequently, defection by partners might not violate the social expectations of individuals with BPD. In contrast, in our experiment, participants with bipolar disorder return the most within the group of individuals with a mental disorder. That is, they report a strong willingness to positively respond to a norm of trust as to signal their partner trustworthiness. Therefore, conditioned on the previous action of the partner, it seems that individuals with BPD are willing to show cooperative behavior. Considering now individuals with high levels of psychopathy, they have been found to make less fair offers, accept less fair offers, and show very high levels of defection^15,16,30^. Major explanations for such behavior point to deficits in emotion regulations (amygdala dysfunctions), which would lead to lack of anxiety, empathy, and guilt, coupled with exaggerated levels of anger and frustration^30^ and to the absence of prepotent biases toward minimizing the distress of others^16^. In this case, our experiments do not confirm those previous results: Indeed, participants with psychosis are the ones who trust, contribute the most to the public good, and are willing to take costly actions to reciprocate their partner’s behavior. It could be possible that, as psychopathic disorders are in fact a large group of different ones, behavioral differences among subgroups may lead to this discrepancy. In connection with these results, it is interesting to note that recent results on a large population of patients with paranoia suggest that distrust is not the best explanation for reduced cooperation and alternative explanations incorporating self-interest might be more relevant^31,32^. This calls for further research into this particular family of MD to clarify whether or not the behavioral characterization applies to all or to a subclass of them.

However, pointing to deficits in social cognition can only account for a partial explanation of individual behavior, and does not contribute to community care narratives. The fact that nothing in this direction has been reported before also reinforces the need to adopt a more holistic view on the interdependencies at play within the mental health collective. Indeed, if statistically relevant differences in cooperative behavior are found across diagnostics, they also depend on the role that actors play in the recovery process. That is, caregivers display exceedingly large degrees of cooperativeness and optimism in one-shot interactions. Caregivers can be thus considered the strong ties of the mental health ecosystem, of particular value when one seeks emotional support. With the de-institutionalization of health systems, caregivers have indeed become key players in care provision. Taking into account their behavior and expectations is therefore of particular interest to extend the support tailored to their needs. These actions should improve the effectiveness of their role by guiding them^33^. Yet, relatives who do not strictly contribute to caregiving practices turn out to be the weak links. It is thus likely that interventions designed to increase their participation in the community might help improve the recovery process.

Also, members of the mental health ecosystem do not equally contribute and benefit from collective action. Rather, systematic behavioral differences arise as the number of social interactions increase, i.e., when teamwork is required for the collective to benefit as a whole. This suggests that considering repeated games may prove extremely insightful for the purpose of the research. Indeed, our experiments show that individuals with MD are the ones who contribute the most to the public good: they make larger efforts towards reaching the collective goal, thus playing a leading role for the functioning of the ecosystem. As a consequence, groups with half or more participants with MD do better in sustaining cooperation in the first rounds, which implies that a community care setting might prove successful for capability building. Yet, large proportions of individuals with MD in a group result in higher inequalities in final gains, which reach the maximum when the number of individuals with MD equals the number of caregivers or relatives. This means that community care perspectives might also take account of group composition to deal with potential inequalities arising from differential capabilities. In summary, we have explored the behavior of all individuals and role groups who make up the mental health ecosystem through an extensive suite of games that simulate strategic social situations. Overall, the results point to the availability of large social capital in the mental health community that can make a difference in the welfare and recovery process of firsthand affected, and suggest that the community-centered approach to mental care may turn out to be very beneficial. Indeed, the behavior of individuals with MD can be better explained by examining not only their cognitive abilities, but also the web of relationships in which they are embedded. Yet, that web of relationships presents opportunities and imposes constraints.

Though we depicted some behavioral differences in dyadic interactions, most importantly we found that individuals with MD show a remarkably larger disposition towards sustaining cooperation within groups. The larger readiness of individuals with MD to contribute to the collective action problem can thus be seen as a way to claim their place in the community. By having participants unaware of their partner’s identity, we could indeed measure participants decisions based solely on the value they placed on the group’s welfare, independently of its composition or other factors. Yet, the fact that participants with MD contribute the most implies for other members of the group lower investments to reach the common target. This, on the other hand, unveils the vulnerability of individuals with a diagnosis of MD. Repeated or periodic and more situated experiments with digital platforms^34^, in the future, can surely provide further valuable insights into the effect of participants prior knowledge of and relation with the partner on their behavior. We are indeed sure that our experimental setup can prove helpful in complementing the diagnostic process of physicians and health professionals and even to evaluate care service providers. On the other hand, other possible application of this approach arises in the realm of behavior change interventions^35^, that should focus on the aspects that are more specific of every disorder.

In conclusion, the results reinforce the idea of community social capital as a key approach to the recovery process based on an ecosystem paradigm (see also the recent results in ref.^36^ about the role and impact of family and community social capital on MD in children and adolescents). Also, if on the one hand the fact that the results of our dyadic games are in general agreement with previous studies validates our procedure; on the other hand it supports the validity and contributions of neuroeconomics and experimental approaches to the study of MD. Finally, given that our work has been carried out in a fully socialized context, this approach can be applied to any similar’ ‘care in the community’ initiative. The adoption of our setup could lead to the identification of core groups that can boost and sustain cooperation within a given community. It can also help in discriminating among different communities in order to identify best practices and optimize resource allocation^37^.

## Methods

All participants were fully informed about the purpose, methods and intended uses of the research. No participant could approach any experimental station without having signed a written informed consent. The use of pseudonyms ensured the anonymity of participants’ identity, in agreement with the Spanish Law for Personal Data Protection. No association was ever made between the participants’ real names and the results. The whole procedure was approved by the Ethics Committee of Universitat de Barcelona. All methods were performed in accordance with the relevant guidelines and regulations.

### Experimental design

As indicated in the main text, the dialogue with the main stakeholders of the mental health ecosystem was at the centre of the project. Around 20 representatives including members of the Catalonia Federation of Mental Health (Federació Salut Mental Catalunya), firsthand affected, relatives, caregivers, and other professionals related to both the health and social sector, informed and validated the whole research through focus groups and further discussions, leading to the largest experiment of this kind ever carried out. Citizen science principles guided the whole experimental design process in order to raise concerns grounded in the daily life of mental health professionals and service users, and to increase public awareness. The experimental dilemmas being proposed served both to advance in knowledge on the social dynamics at play within ‘care in the community’ settings and as a self-reflection experience for all participants. The experimental design process developed in four main phases: (i) identification of the behavioral traits perceived as of fundamental importance within the community, (ii) operationalization of those same behavioral traits thorugh game theoretical paradigms and literature reviews, (iii) definition of the socio-demographic information relevant for the analysis, and (iv) a beta testing of the digital interface (including contents, time duration, and language used). The locations where the experiments took place were accorded with the Catalonia Federation of Mental Health in an attempt to explore the functioning of some communities of interest for inclusive and effective policy making. The Federation provided a fundamental support throughout the whole experiments’ implementation, serving as a crucial intermediaire between the scientists and different mental health collectives. It also provided valuable insights to better interpret the data obtained.

### Participants and procedure

To our knowledge, experimental work on this issue has been conducted only recently and on specific collectives of orders of magnitude smaller. A total of 270 individuals participated in the experiments, that were run over 45 sessions between October 2016 and March 2017. The experiments were carried out in Girona (n = 60), Lleida (n = 120), Sabadell (n = 48) and Valls (n = 42). Participants were either diagnosed with a mental condition (n = 169) or members of the broader mental health ecosystem (n = 101), including professionals of the health and social sector (n = 52), formal and informal caregivers (n = 17), relatives (n = 9), friends (n = 4) and other members of the collective (n = 19). Individuals with a mental condition had to self-assess their diagnosis selecting one from a spectrum of options agreed upon with representatives of the mental health ecosystem during the co-design phases of the experiment. Those participants who had received more than one diagnosis had to select the one they considered to be the most relevant. Overall, they had received a diagnosis of psychosis (n = 63), depression (n = 33), anxiety (n = 31), bipolar disorder (n = 17) or other unspecified diagnosis (n = 25). They ranged in age from 21 to 77 years old (these are weighted values since for ethical and privacy reasons participants were only asked to choose among different age ranges) with 47.2 years on average. Further, 55.6% were men and 44.4% were women. Yet, actors involved in the recovery process were predominantly women (76.2%), and up to 21.8% of them was over 60 years old (see Supplementary Section 1.1). Participants were told that they would play against each others a set of games meant to explore human decision-making processes. They played in random groups of six players through a web interface specifically developed for the research. They were informed that they had to make a decision under different conditions and against different opponents in every round. Every game represented an interactive situation requiring the participants to make a decision, the result of which depended also on the opponent’s behavior. To incentivize the participation, they would earn a voucher worth their final score (the experimental settings and instructions, can be found in the Supplementary Section 1.2 and 1.3 respectively). First, participants participated in a Collective Risk Dilemma^23^ against five opponents. Briefly, the game is a public goods game with threshold: If the participants’ total contribution after 10 rounds is lower than a given threshold, they loose all the money they kept with a probability of 90%. Otherwise, they are told that the money collected in the common fund are spent in reforesting land plots in Catalonia, where the experimental sessions took place, and each participants earns the money left in the personal account. After completing the task, participants played one round of the Trust Game^38^ in both roles: as trustors and as trustees. They played against different partners in each role. Finally, they played one round of a Prisoner Dilemma^39^ with (unincentivized) belief elicitation about their counterpart’s behavior prior to playing. Before starting the games, participants had to complete a brief survey covering some key dimensions of their sociodemographic background. The assignment of players’ partners in the dyadic games was completely random and every action was made with a different partner. The average (standard error of the mean) time for completing the three experiments (CRD, PD and TG and tutorials) is around 12 minutes, 705.86 ± 17.93 s. At the end of each session, participants received a gift card worth their earnings. The average individual earning is 46.84 ± 0.77 MUs equivalent to a 4.04 ± 0.077 EUR voucher. The behavioral patterns that emerged do not reveal significant variation across the different experiments, which may suggest that our results are robust to generalizations (see Supplementary Section 1.7).

### Statistical analysis

Results were analyzed at two levels: first, we tested for behavioral differences between the whole group of individuals with mental condition compared to members of the mental health ecosystem; we then checked for systematic behavioral variation across diagnostics and role played in the recovery process. In one shot, two-person dyadic interactions we performed Mann-Whitney-U tests for independent groups to compare the distributions of cooperative choices (PD), and initial and back transfers (TG), between individuals with and without a mental condition. We then checked for marginal differences within groups using Kruskal-Wallis tests, and post-hoc comparisons were run with Mann-Whitney-U tests adjusting for p-values with the Holm-Bonferroni method. Welch’s two-tailed t-tests were performed to check for differences in average contributions (CRD) between participants with and without a MD, controlling for unequal variances and sample sizes. Finally, ANOVA and further Tukey HSD post-hoc comparisons served to check for differences in average contributions over round across diagnostics and members of the mental health community.

### Accession codes

Data is available in an structured way at Zenodo public repository with DOI 10.5281/zenodo.1175627.

## Electronic supplementary material

Supplementary Information
