# Author Correction: Quantitative account of social interactions in a mental health care ecosystem: cooperation, trust and collective action

**DOI:** 10.1038/s41598-018-32562-4

**Published:** 2018-09-26

**Authors:** Anna Cigarini, Julián Vicens, Jordi Duch, Angel Sánchez, Josep Perelló

**Affiliations:** 1 Departament de Física de la Matèria Condensada, Universitat de Barcelona, 08028 Barcelona, Spain; 2 Universitat de Barcelona Institute of Complex Systems UBICS, 08028 Barcelona, Spain; 3 Departament d’Enginyeria Informàtica i Matemàtiques, Universitat Rovira i Virgili, 43007 Tarragona, Spain; 4 Northwestern Institute on Complex Systems (NICO), Northwestern University, 60208 Evanston, IL USA; 5 Grupo Interdisciplinar de Sistemas Complejos (GISC), Unidad de Matemática, Modelización y Ciencia Computacional, Universidad Carlos III de Madrid, 28911 Leganés, Spain; 6 Unidad Mixta Interdisciplinar de Comportamiento y Complejidad Social (UMICCS) UC3M-UV-UZ, Universidad Carlos III de Madrid, 28911 Leganés, Spain; 7 Institute UC3M-BS of Financial Big Data, Universidad Carlos III de Madrid, 28903 Getafe, Spain; 8 Instituto de Biocomputación y Física de Sistemas Complejos (BIFI), Universidad de Zaragoza, 50009 Zaragoza, Spain

Correction to: *Scientific Reports* 10.1038/s41598-018-21900-1, published online 28 February 2018

This Article and the accompanying Supplementary Information contains a repeated error where ‘Prisoner’s Dilemma’ and its acronym ‘PD’ should read ‘Hawk-Dove game’ and ‘HD’.

Additionally, in the Discussion section

“In this case, our experiments do not confirm those previous results: Indeed, participants with psychosis are the ones who trust, contribute the most to the public good, and are willing to take costly actions to reciprocate their partner’s behavior.”

should read

“In our experiments participants with psychosis, a mental condition which is unrelated to psychopathic impairments, are the ones who trust, contribute the most to the public good, and are willing to take costly actions to reciprocate their partner’s behavior.”

