# Supplementary material for: Quantitative account of social interactions in a mental health care ecosystem: cooperation, trust and collective action
Source: Sci Rep. 2018 Feb 28;8:3794. doi: 10.1038/s41598-018-21900-1 (PMC5830605; doi:10.1038/s41598-018-21900-1)
Supplement: Supplementary file 1 — Supplementary Information [file 41598_2018_21900_MOESM1_ESM.pdf]

Supplementary Information

Quantitative account of social interactions in a mental health care ecosystem: cooperation, trust and collective action

Anna Cigarini,[1,2] Julián Vicens,[1,2,3] Jordi Duch,[3,8] Angel Sánchez,[4,5,6,7] and Josep Perelló[1,2,⋆]

[1]Departament de Física de la Matèria Condensada,
Universitat de Barcelona, 08028 Barcelona, Spain

[2]Universitat de Barcelona Institute of Complex Systems UBICS,
Universitat de Barcelona, 08028 Barcelona, Spain

[3]Departament d'Enginyeria Informàtica i Matemàtiques,
Universitat Rovira i Virgili, 43007, Tarragona, Spain

[4]Grupo Interdisciplinar de Sistemas Complejos (GISC),
Unidad de Matemática, Modelización y Ciencia Computacional,
Universidad Carlos III de Madrid, 28911 Leganés, Spain

[5]Unidad Mixta Interdisciplinar de Comportamiento y Complejidad Social (UMICCS),
Universidad Carlos III de Madrid, 28911 Leganés, Spain

[6]Institute UC3M-BS of Financial Big Data,
Universidad Carlos III de Madrid, 28903 Getafe, Spain

[7]Instituto de Biocomputación y Física de Sistemas Complejos (BIFI),
Universidad de Zaragoza, 50009 Zaragoza, Spain

[8]Northwestern Institute on Complex Systems (NICO),
Northwestern University, 60208 Evanston, IL, USA

January 15, 2018

Contents

1 Supplementary Notes **3**
1.1 Sociodemographics . . . 3
1.2 Experimental settings . . . 3
1.3 Experimental instructions . . . 4
1.3.1 Initial instructions . . . 4
1.3.2 Sociodemographic questionnaire . . . 5
1.3.3 Tutorial . . . 5
1.3.4 Game One: The Climate Game . . . 6
1.3.5 Game Two: Investor Game . . . 6
1.3.6 Game Three: Prize Game . . . 7
1.3.7 Results . . . 8
1.4 Cooperation and Optimism in the Prisoner's Dilemma . . . 8
1.5 Trust and Reciprocity in the Trust Game . . . 9
1.6 Collectivity in the Collective-Risk Dilemma . . . 10
1.6.1 Game evolution . . . 11
1.6.2 Effects of relation with firsthand affected . . . 11
1.6.3 Effects of group composition . . . 11
1.6.4 Inequalities . . . 12
1.6.5 Earnings and Contributions . . . 12
1.7 Robustness to generalizations . . . 12

2 Supplementary Figures **13**

3 Supplementary Tables **26**

1 Supplementary Notes

1.1 Sociodemographics

Supplementary Fig.S1 summarizes some key background figures of our 270 participants. It is noteworthy that although anybody aged over 18 could participate in the experiment, only a small fraction (7%) of participants with a mental disorder were under 30 years old. This implicitly suggests that younger individuals with mental disorders may not be well represented within mental health communities. Also, few were working (20%) and most were not in a stable relationship (55%), with more than half with primary or no education (53%). On the other hand, actors involved in the recovery process were more heterogeneously distributed across age groups. Yet, only 28% were either married or in a civil union, and 43% were not in a paid job.

1.2 Experimental settings

All experimental sessions were carried out in the infrastructures provided by the Catalan Federation of Mental Health. These include: the World Mental Health Day event, organized in Lleida on October 8th 2016 in the background of the Old Cathedral of Lleida. Two social events promoted and arranged by the Federation as part of the yearly social activity plan: in an assembly hall in Valls on March 18th, 2017, and in a community mental health center in Sabadell on March 27th, 2017. And a local employment insertion centre for people with mental disorders in Girona on March 24th, 2017. The participants played in random groups of six players each, for a maximum of three groups at a time depending on the location's constraints. Experimental stations were spatially arranged so that participants could not see each other. Also, they were rigorously prevented from talking or signaling one another. To further guarantee that potential interactions among players sitting close to each other did not influence the results of the experiment, the assignment of players' partners was completely random. All of the participants played through a web interface specifically developed for the experiment. The participants were shown a brief tutorial, but were not given any clue. They were informed that they had to make decisions under different conditions and against different opponents in every round. Also, to incentivize their participation, participants were told that they would earn a voucher worth their final score. We made sure the interface be the most simple and understandable to ensure the correct understanding of the tasks. Also, the interface was the same for everybody. We made sure to avoid the research be upsetting or harmful for the participants by presenting the experiment as a game and playful activity. Three to four researchers closely monitored each session to guarantee the experimental protocol be strictly followed. Yet, the researchers provided help when required.

1.3 Experimental instructions

Participants were given the following instructions (translated from the original Catalan and Spanish online versions).

1.3.1 Initial instructions

Screen 1

Welcome to Games for Mental Health

We propose you to participate in a game which is both an experience and a scientific inquiry. The data collected are part of a participatory research which combines together the interests of scientists, physicians, families, individuals with a mental condition, and social workers among others.

We want to understand with you how do we behave in a number of everyday situations. There is no right or wrong answer. Ah! Do not forget: You will be playing with other people, not with a robot.

Yours and the other player's personal interests will be at odds. As it does in number of decisions you make everyday when you head down the street. No more, no less.

Are you ready? It will only take 10 minutes.

Screen 2

Pick a pseudonym

You will need it at the end of the game to claim a prize, keep it in mind! The data collected are anonymous, the pseudonym prevents the identification of the players. Avoid whatever references to your personal identity.

Screen 3

Legal note

Personal anonymized data provided through the registration process and collected for Games for Mental Health project, will be included in the repository 'Proyectos de investigación, desarrollo e innovación con datos de carácter personal de nivel básico', property of the University of Barcelona. The data will be processed in accordance with the objectives set up in the aforementioned project. You can at any time exercise the rights of access, rectification and withdrawal from the project. For this purpose you are required to forward a written notification, together with a copy of your DNI or any other identity document, to Secretaria General de la Universidad de Barcelona, Gran Via de les Corts Catalanes 585, 08007 Barcelona; or via email at secretaria.general@ub.edu.

I have read the above terms and conditions.

1.3.2 Sociodemographic questionnaire

Screen 4

Enter the following data

Question 1: *Why are you being looked after?* Answer 1: *Depression* Answer 2: *Bipolar disorder* Answer 3: *Psychosis (schizophrenia, etc.)* Answer 4: *Anxiety* Answer 5: *Other mental disorders* Answer 6: *I do not have any mental disorder*

Screen 4b

Enter the following data (IF Screen 4: 'I do not have any mental disorder')

Question 2: *Are you?* Answer 1: *Non-caregiving relative* Answer 2: *Caregiving relative or informal caregiver* Answer 3: *Friend* Answer 4: *Professional (health sector, social sector, etc.)* Answer 5: *Others*

Screen 5

Enter the following data

Question 3: *Employment status* Answer 1: *Not working* Answer 2: *Working* Answer 3: *Working in CET (Centro Especial de Trabajo)*

Question 4: *Gender* Answer 1: *Man* Answer 2: *Woman*

Question 5: *Enter your age*

Question 6: *Enter your Postal Code*

Screen 6

Enter the following data

Question 7: *Civil status* Answer 1: *Single* Answer 2: *Married* Answer 3: *Civil union* Answer 4: *Divorced* Answer 5: *Widow* Answer 6: *Others*

Question 8: *Educational level* Answer 1: *None* Answer 2: *Primary education* Answer 3: *Secondary education* Answer 4: *Baccalaureate or equivalent* Answer 5: *Professional education* Answer 6: *University studies* Answer 7: *Not specified*

1.3.3 Tutorial

Screen 7

Tutorial: How to play

(Screenshots in Supplementary Fig.S10)

The activity you are about to engage consists of a set of games that you will be presented. It is extremely important that during the experiment you DO NOT TALK with other players. You are not expected to behave in any particular way: there are no right or wrong answers. If you

*exit the game before its end, you won't be able to enter again! The money you will get at the end of the game will depend on the decisions you will make throughout the experiment. *Use the side arrows to move through the tutorial. Once you are done, you will start with the first game.*

1.3.4 Game One: The Climate Game

(Screenshots in Supplementary Fig.S11)

In this game you will simultaneously play with 5 PLAYERS. Each player will be endowed with 40 MONETARY UNITS. The objective is to collect 120 MONETARY UNITS in a common fund to promote actions against climate change.

The game consists of 10 rounds. In each round every player has to contribute between 0 and 4 monetary units of her actual endowment to the common fund. You have 20 seconds to make your decision, or else the computer will do it for you. If you run out of time for 2 or more rounds your earnings will be 0 in this game.

At the end of each round you will be presented with a summary containing information on: 1. The total amount of money collected in the common fund. 2. The contribution of every player in the previous round. 3. The initial and current endowment of every player.

If, after 10 rounds THERE ARE 120 OR MORE MONETARY UNITS in the common fund: THE MONETARY UNITS YOU SAVED will add on a voucher for ABACUS shop. We will support actions to promote reforestation in Catalunya.

Example: The game ends and the whole group contributed 130 monetary units. If you contributed with 26 monetary units out of your initial 40, you will earn a voucher with the equivalent of the remaining 14 monetary units!

If, after 10 rounds THERE ARE LESS THAN 120 MONETARY UNITS in the common fund: 1.With a 10% probability you will earn a voucher for ABACUS shop with the equivalent of the MONETARY UNITS YOU SAVED. 2.We will not be able to promote actions for reforestation.

Example: The game ends and the whole group contributed 94 monetary units. If you contributed with 12 monetary units out of your initial 40, you will earn a voucher with the equivalent of your remaining 28 monetary units only with a 10% probability. In 90% of cases you will not earn anything.

1.3.5 Game Two: Investor Game

(Screenshots in Supplementary Fig.S12)

You are faced with a good dilemma. You can be either an investor who has to decide whether to invest in a new business. Or you can happen to be an entrepreneur who needs money for his enterprise. What role will you play? What are you going to do? Think carefully about which decision to take in every situation.

You are: The investor! Your game partner is an entrepreneur asking you to invest in his enterprise. With the money you give him he will start a business which will earn him THREE TIMES your investment. In this game you earn the money you will not invest plus the money that the entrepreneur will return you. He will have to decide which portion of the money earned to return you, but has NO OBLIGATION to reciprocate. How much money do you want to invest in the business?

You are: The investor!
You invested a total of...
And the entrepreneur returned you...
You earn...
And the entrepreneur earns...
Thank you very much for participating! Ready for the next game?

You are: The entrepreneur! Your game partner is an investor with 10 MONEY UNITS, and has to decide how much money to invest in your business. The INVESTOR decided to invest in your business: ... Thanks to the investment you earned: ... Which portion of the money earned do you want to return him?

You are: The entrepreneur!
The investor invested a total of...
And decided to return...
The investor earns...
And you earn...
Thank you very much for participating! Ready for the next game?

1.3.6 Game Three: Prize Game

(Screenshots in Supplementary Fig.S13)

You and the other player received a prize worth 10 MONEY UNITS each. Now you both need to make a decision which will affect what you both will earn: KEEP the prize or MULTIPLY it, taking part of the prize of the other player. It is not that easy at it seems. According to what the other player does you might not be earning anything.

The rules are as follows:
If you choose KEEP and the other chooses KEEP: You earn 10 and she earns 10.
If you choose KEEP and the other chooses MULTIPLY: You earn 5 and she earns 15.
If you choose MULTIPLY and the other chooses KEEP: You earn 15 and she earns 5.
If you choose MULTIPLY and the other chooses MULTIPLY: You earn 0 and she earns 0.
Is everything clear? If not, raise your hand.

Before playing the game... A CONFESSION: What do you think the other player will do?
Now it's your turn... What do you want to do with your prize?

You chose... You earn... She chose... She earns...

1.3.7 Results

Thank you for participating in all games!
Here is a summary of what you have earned in each game:
Climate Game:...
Investor Game:...
Entrepreneur Game:...
Prize Game:...
Total money:...
You have earned a voucher Abacus worth: ...

You can register at JocXlaSalutMental.org to receive some interesting preliminary results. Do not forget to claim your prize with your pseudonym.

1.4 Cooperation and Optimism in the Prisoner's Dilemma

Participants played a one-shot Prisoner's Dilemma (PD) with one random partner. They had to simultaneously choose to keep a prize (namely cooperate) or to multiply it (namely defect). No matter what the other does, defection yields a higher payoff than cooperation. The dilemma is that if both defect, both do worse than if both had cooperated. We define cooperation as the number of cooperative decisions over the total number of decisions. Before making the choice, participants were asked whether they believed the partner would cooperate or defect. Intuitively, believing that the opponent is going to cooperate involves hopefulness and confidence about the future, especially in interactions with uncertain consequences. We thus defined optimism as the number of times participants expected cooperation over the total number of expectations elicited. Supplementary Figure S2 illustrates the joint distribution of participant cooperation and their beliefs about the partner's cooperation.

We tested whether estimates about the partner's decision do influence behaviour against the hypothesis that individuals act independently of what they expect other people do. We performed multiple Chi-squared tests to check for associations between expectations of cooperation and cooperative behaviour across groups. As Supplementary Figure S2 illustrates, the relation turns out to be significant for individuals with MD ($\chi^2 = 17.442$, p<0.0001) and caregivers ($\chi^2 = 7.6604$, p = 0.005645) (Top), and for participants with psychosis ($\chi^2 = 8.422$, p= 0.003707)(Bottom). We also compared all predictions with the observed partner's decision to see whether participants guess right. Overall, 69% of predictions are correct.

However, no significant differences are found in the frequency of cooperative interactions if we differentiate participants only by having or not a mental condition ($c = 0.58 \pm 0.04$ and $c = 0.65 \pm 0.05$ respectively). The distribution of cooperative choices for the two groups is indeed statistically equivalent ($\chi^2 = 1.143$, p = 0.28). This also applies when participants' beliefs about the partner's behaviour are elicited ($\chi^2 = 0.12705$, p = 0.72). Expecting the partner to cooperate or to defect does not significantly varies between participants with and without a mental condition ($c_{\text{exp}} = 0.57 \pm 0.04$ and $c_{\text{exp}} = 0.6 \pm 0.05$, respectively). Also, if cooperation varies across diagnostics and role groups, expectations do not. A Kruskal-Wallis test could not reject the null hypothesis of equality of distributions (Kruskal-Wallis, H= 2.3112, p = 0.3149). For further details about cooperation and expectation rates across groups see Table S1 and S2 respectively. For average cooperation and expectation across groups see Table S5.

1.5 Trust and Reciprocity in the Trust Game

Participants' played one round of the Trust Game in both roles: as senders and as returners, against different partners. They were given 10 Monetary Units (MUs) and had to decide how much money to send to their anonymous opponent between 0; 2; 4; 6; 8; or 10 MUs. Then, they had to decide how much of the total received to send back: either 0; 25%; 50%; 75% or 100% of the amount received and tripled by the experimenter.

The initial transfer captures player's intention to trust the opponent in the hopes of receiving the same amount of money (or more) in return. Yet, trusting involves risks in that the opponent has no obligation to reciprocate and to retain all the money earned for himself. Therefore, trusting may result in a smaller gain for the sender. We define trust as the amount of MUs transferred by the investor, normalized between 0 and 1. On the other hand, the back transfers of the returner provides with a baseline measure of reciprocity, which has to do with repaying in kind or rather punishing the partner's behavior. However, the decision to reciprocate trust also cause a reduction in the returner's gain. It thus depend on the offset between maximizing personal gains relative to the appreciation of the trust that was given. We defined reciprocity as the amount of MUs returned as a portion of the amount available to return. Participants were aware that they would play both roles with different partners.

Overall, most participants trust 6 MUs (31.8%) or 4 MUs (28.5%). Only 4 out of 270

participants send nothing, and as many as 15% transfer all their endowment to their anonymous opponent, the majority of whom (69%) has MD. The modal initial transfer of individuals with MD is 6 MUs trusted, while that of actors involved in the recovery process is 4 MUs. As for back transfers, most participants (41%) reciprocate with 50% MUs, that is they split what they received in half. However, as many as 39% participants return 25% of the amount available to return. Also, 7% participants keep all for themselves (67% of them belong to the MD group). Yet, 4% participants return everything, and almost all of them (92%) has a mental disorder. This implicitly suggest that untrustworthy behavior is more prevalent among participants with MD, yet they are more likely to return everything compared to caregivers and non caregivers. Further, while the modal behavior of individual with MD is to reciprocate with 25% MUs, the modal return transfer of individuals without MD is 50% MUs.

Supplementary Figure S3 aggregates results for individuals with MD, caregivers and non caregivers (Top) and for each diagnostic (Bottom). As it can be seen from the plot, on average, all participants trust more than half of their endowment, and the distribution of initial transfers is similar across groups. No variation is indeed found in trust levels between participants with MD, caregivers and non caregivers (Kruskal-Wallis, H= 2.7457, p= 0.2534). Also, all participants return, on average, positive amounts. However, some minor variation is found in back transfers across diagnostics, as mentioned in the letter, see Table S3 and S4. For average initial and back transfers across groups see Table S6.

1.6 Collectivity in the Collective-Risk Dilemma

Participants played in group of six players a Collective-Risk Dilemma over 10 rounds. They were endowed with 40 MUs and asked, in each round, to simultaneously contribute to a common fund with 0, 2, or 4 MUs. They had 20 seconds to made their decision after which the computer would make it for them. At the end of each round they would receive information about: the amount of money collected by the group members over the earlier rounds; individual and group members contributions in the previous round; and individual retained MUs. They were told that if the group's total contributions at the end of all rounds reached or surpassed a target amount set at 120 MUs, a certain collective action to mitigate climate change would be promoted and all group members would gain their individual retained funds. If insufficient contributions were made, the contributors would loose their contributions with 90% probability, and the collective action would not be promoted. A social dilemma arises in that everybody benefits from reaching the target sum, but players are tempted to contribute 0 and to benefit personally at the expenses of other group members. The Collective-Risk Dilemma is the paradigm that best captures the social dilemma that emerges from the conflict between group and individual interests. It allows for the realistic modelling of group interactions and individual sacrifice for the group's welfare. Indeed, the more a participant invests in the collective good, the higher the probability that the group reaches the target sum. Yet, the less money remains in his or her personal account. In

contrast, failure to reach the target sum implies a high risk that the remaining money in the personal account will be lost. At the same time, the more others invest, the less a subject needs to invest for the group still to reach its target sum. We thus define collectivity as individuals' contribution to the group's welfare. We measure it as the portion of the amount contributed to the common fund over rounds out of their initial endowment (40 MUs).

1.6.1 Game evolution

Groups of six participants contributed in the common goal during 10-rounds with the objective to collect 120 MUs at the end of the game. As we observe in Supplementary Fig.S4, the average contribution of the groups of six in each round is above the fair contribution during all the game, therefore in average the groups always are in disposition to achieve the target. We can observe how in groups with different proportion of participants with MD the trend doesn't change.

1.6.2 Effects of relation with firsthand affected

We study the effects of contribution in relation of participants affected with MD. We can observe the average contribution and the evolution of contribution over round among participants affected and not affected. We observe significant differences between participants with MD and the rest of the collective. However, within the group of participants with MD we observe no significant differences in their contributions (see Supplementary Fig.S5). The most of participants with MD (43.6%) selected the maximum contribution (4), while the caregivers (46.5%) and non-caregivers (48.9%) mostly selected the fair contribution (2).

1.6.3 Effects of group composition

Each game was composed of participant with different roles in the ecosystems (affected with MD, caregivers and non-caregivers), we analyze if the distribution of participants with different roles has an effect on the collective contribution.

The evolution of the game (Supplementary Fig.S6), in which the most of participants are affected with MD and games with the most of participants are not affected, differs (t-test $p < 0.01$) in the first part of the game (rounds 1-5). Nonetheless average contribution in the last rounds has not significant differences (t-test $p > 0.05$).

The average contribution of participants with MD are 2.36 MU and 2.35 MU in the two contexts, with and without a majority of MD, is greater than the average contribution of the rest of collectives 2.09 MU and 2.10 MU (Supplementary Fig.S7). In average, the contribution based on role doesn't differ depending of the composition of the group.

1.6.4 Inequalities

Participants affected with mental disorder tend to contribute more than the others in the collective-risk dilemma. This behaviour create inequalities, the most contribute the less earnings at the end of the game, we mesuare the inequalities created by this behaviour using the Gini index. We calculate the index in games with different proportions of participants with MD, and observe how in games with the most of participants affected with MD the inequalities increase, specially in the case with half of participants affected.

1.6.5 Earnings and Contributions

Final earnings include all participants (270). However, participants who did not contribute in two or more rounds, and had the computer contributing for them, did not get any profit. Their final earning is 0. The average (mean±sem, standard error of the mean) earnings of all subjects is 16.50 ± 0.53 MUs (see Supplementary Fig. S9 for final earnings across groups).

Individual contributions per round (Supplementary Fig.S8) includes all participants (270). Yet, contributions in rounds where the computer selected for the participant were not included in the analysis. On the other hand, group contributions include both contributions made by the participants and computer selections.

1.7 Robustness to generalizations

We tested for differences in game behaviors across the four experiments, namely Lleida, Girona, Sabadell and Valls experiments. No significant variation was found in the frequency of cooperative behaviors (Kruskal-Wallis, H= 2.3827, p = 0.4969), general expectations of cooperative behavior (Kruskal-Wallis, H= 0.37976, p = 0.9444), initial and back transfers in the TG (Kruskal-Wallis, H= 2.6683, p= 0.4456, and H= 3.0222, p= 0.3882), nor contributions to the common good (ANOVA, F= 0.21, p= 0.647).

2 Supplementary Figures

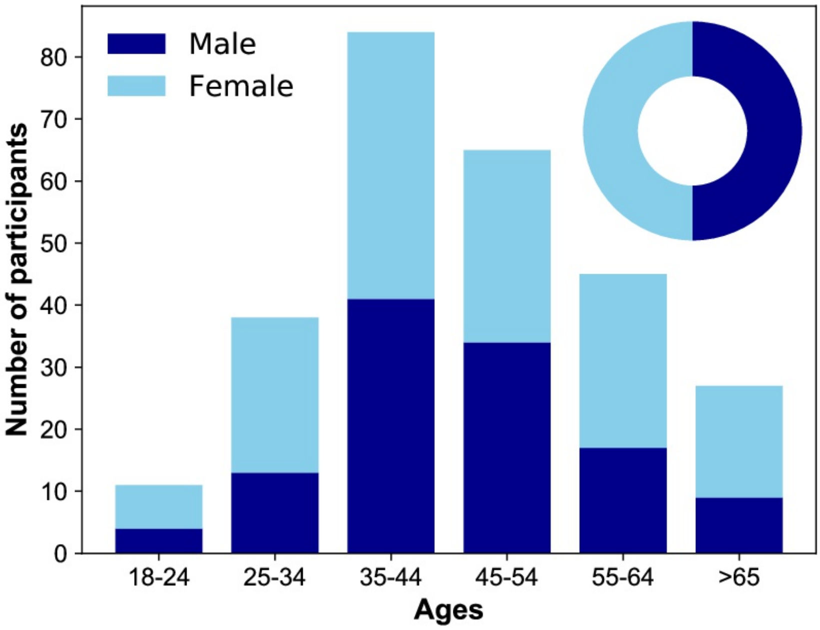

Supplementary Figure S1: Sociodemographics. Age and gender distributions. Age ranges are those given in the survey following Ethics and Privacy committe advice. There were 270 participants: 55.6% were men and 44.4% were women.

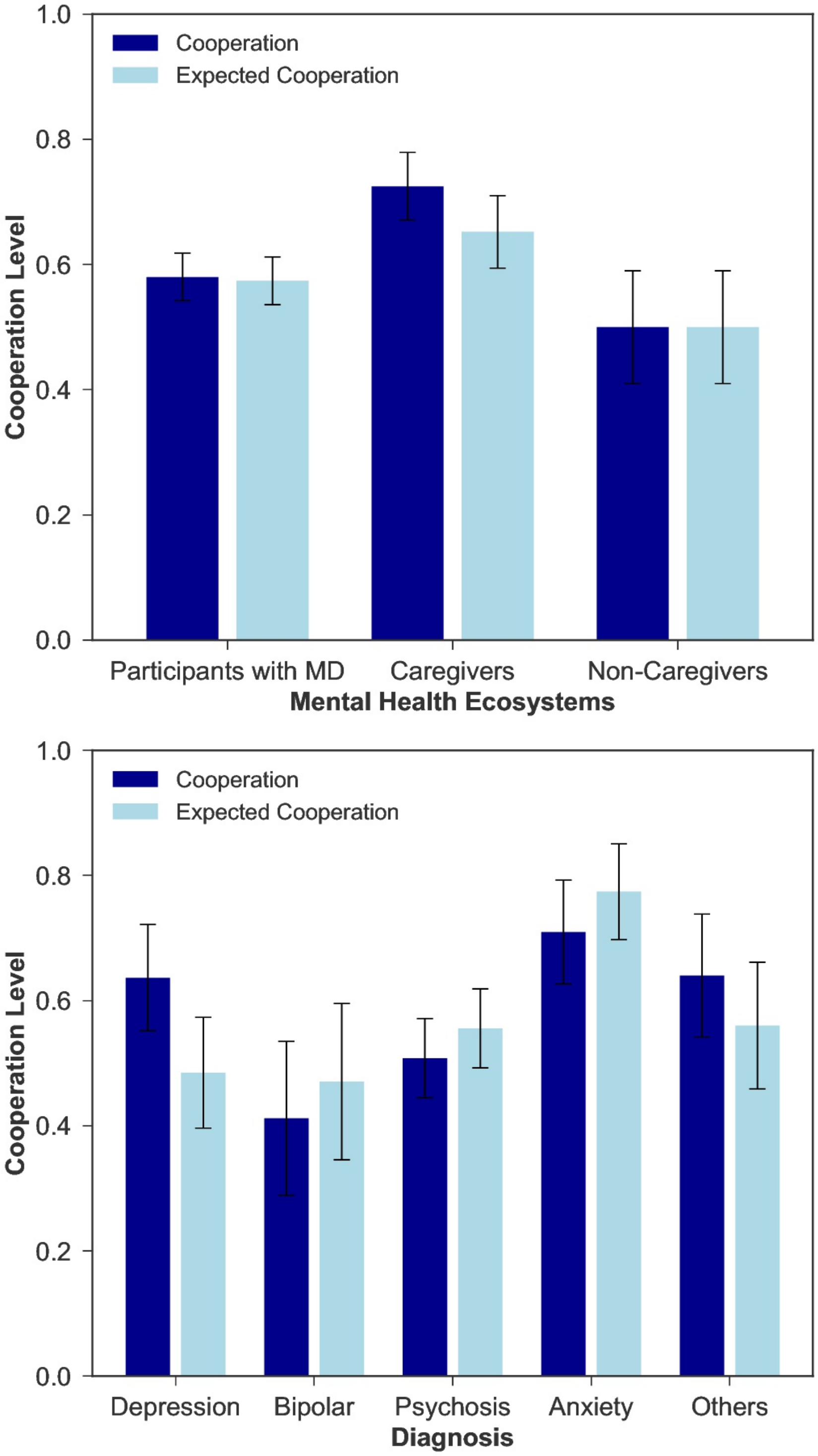

Supplementary Figure S2: Association between cooperation and expected cooperation in the Prisoner's Dilemma. The figure displays the frequency (mean and standard error of the mean) of cooperative choices and expected cooperation across groups (Top) and diagnostics (Bottom). Chi-squared tests for association between expectation and cooperation are significant for individuals with MD (χ^2 = 17.442, p<0.0001) and caregivers (χ^2 = 7.6604, p $<$ 0.01) (Top), and for participants with psychosis (χ^2 = 8.422, p$<$ 0.005)(Bottom).

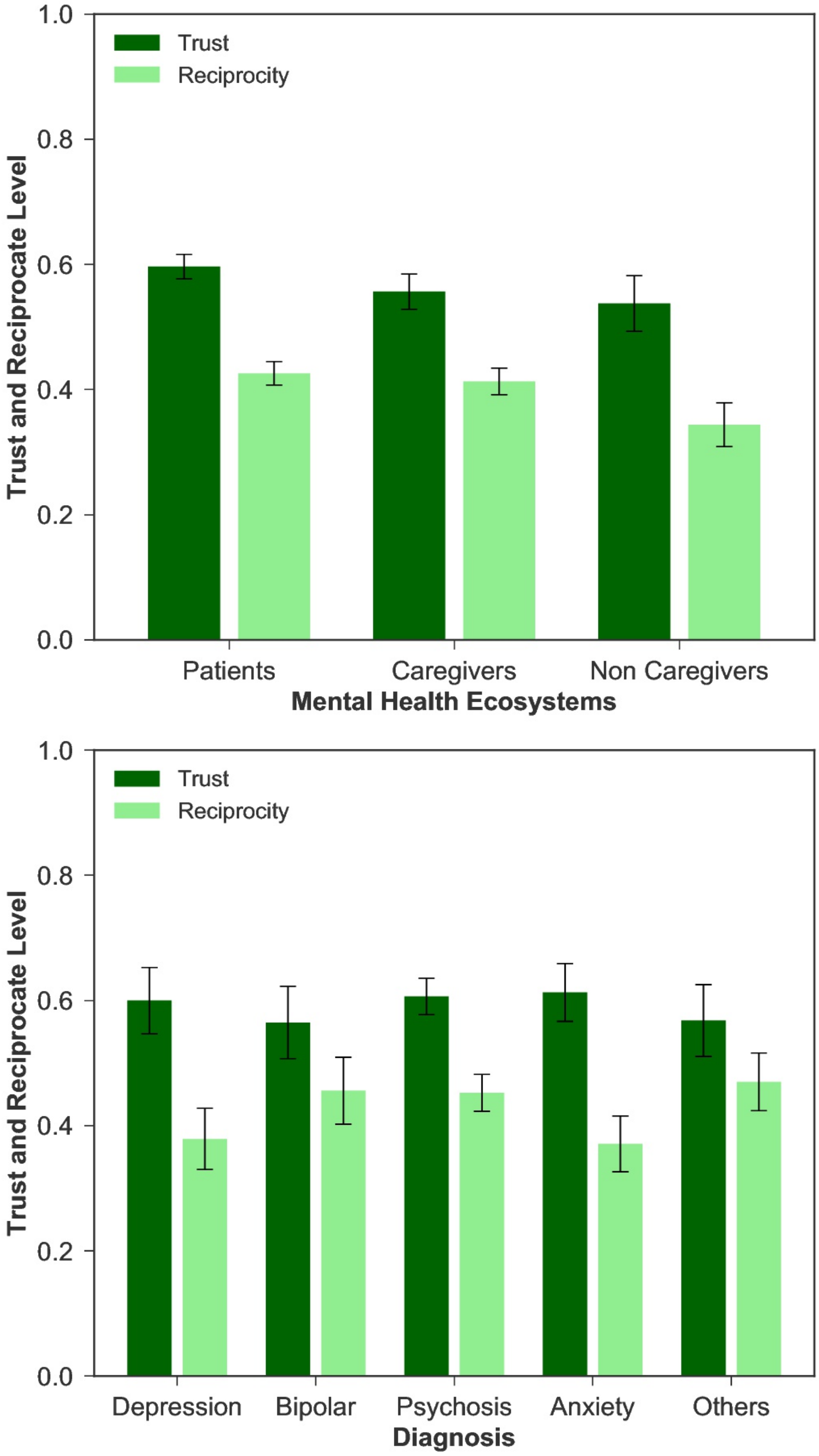

Supplementary Figure S3: Trust and Reciprocity levels in the Trust Game. Initial and back transfers (mean and standard error of the mean) in the TG across groups (Top) and diagnostics (Bottom).

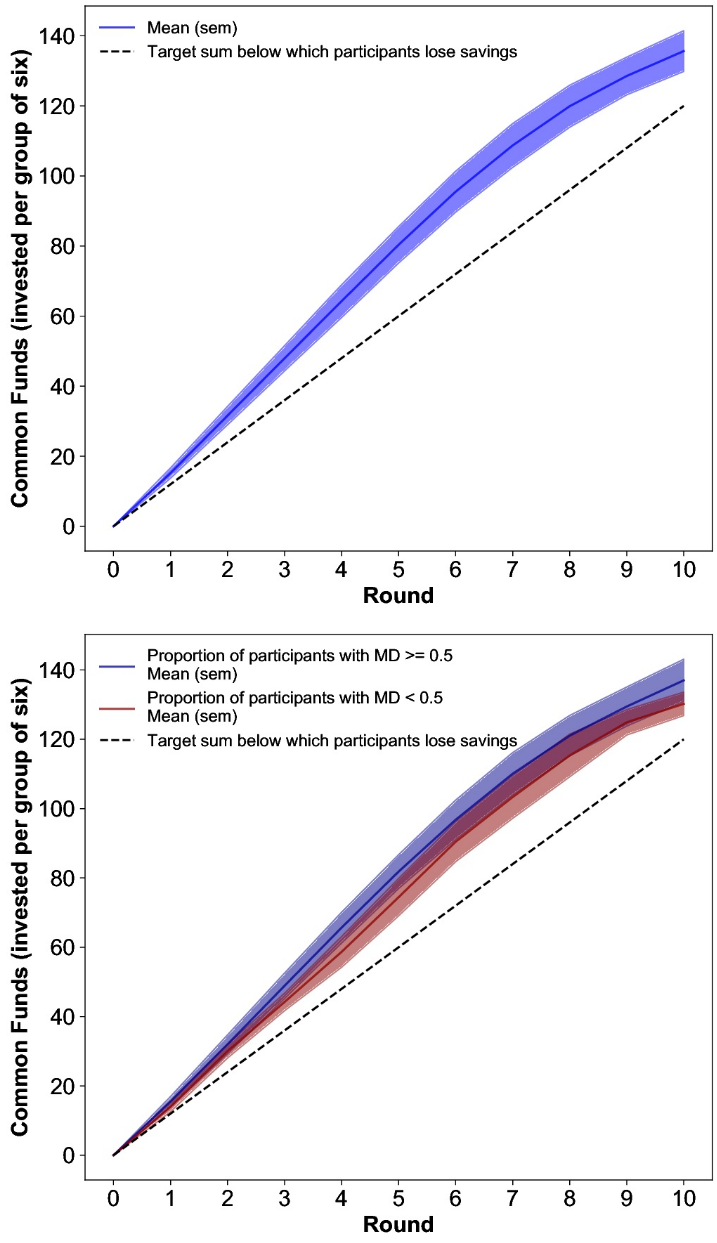

Supplementary Figure S4: Evolution of contributions during the games. (Top) Evolution of aggregate contributions (mean and standard error of the mean) to the common fund over rounds. (Bottom) Evolution of aggregate contributions (mean and standard error of the mean) over rounds depending on the portion of firsthand affected within groups.

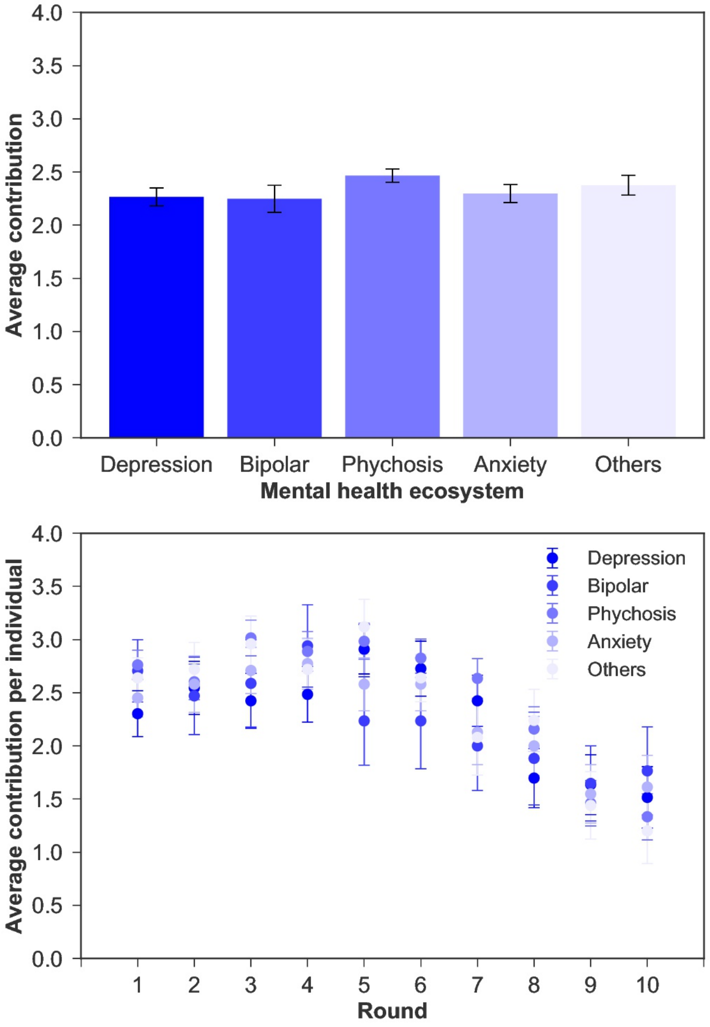

Supplementary Figure S5: Contributions across diagnostics. (Top) Average aggregate contributions (mean and standard error of the mean) and (Bottom) evolution of contributions (mean and standard error of the mean) over rounds.

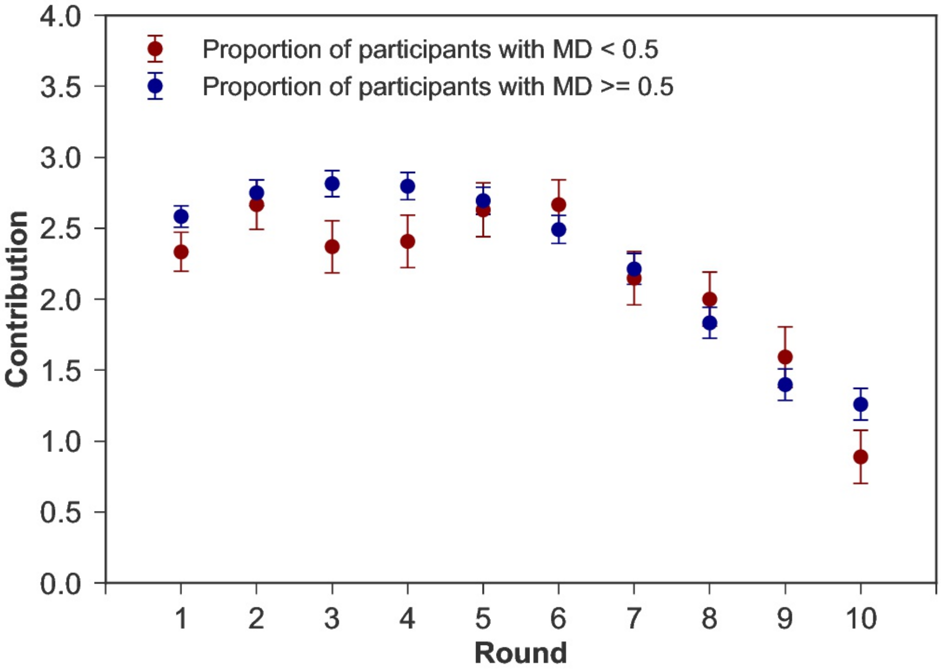

Supplementary Figure S6: Contributions by group composition. Evolution of contributions (mean and standard error of the mean) over round according to the portion of participants with MD within groups.

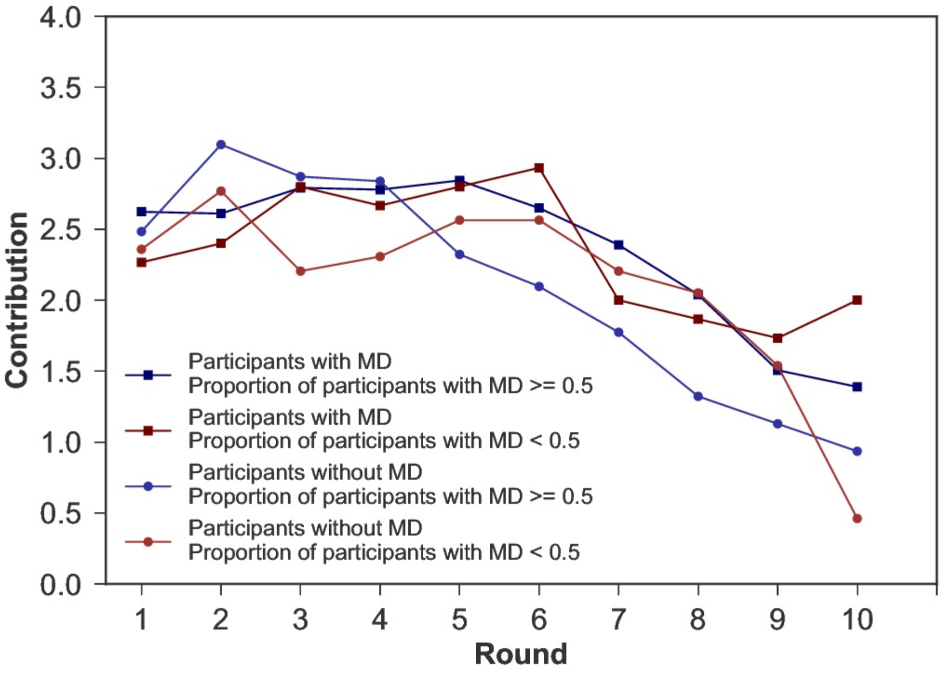

Supplementary Figure S7: Contributions by group composition and role. Evolution of individual contributions over round of individuals with and without a mental condition by portion of individuals with MD within groups.

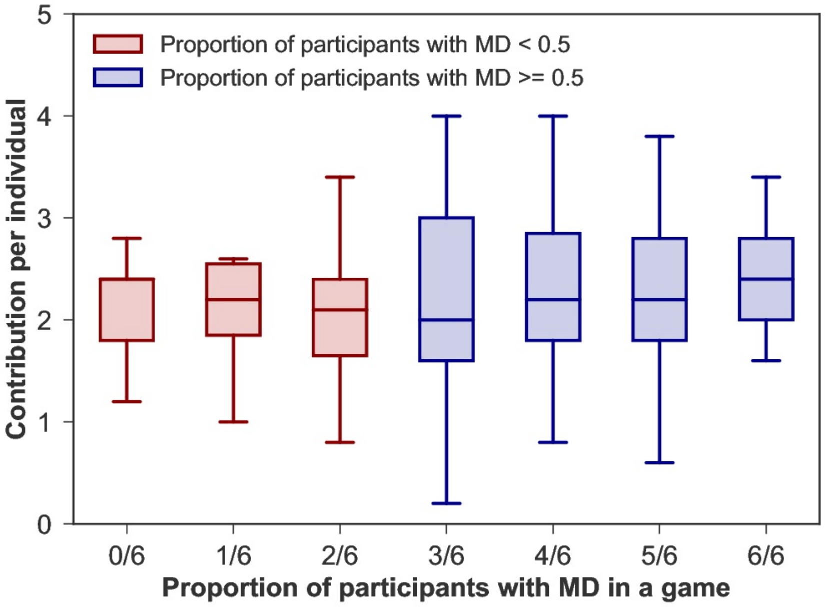

Supplementary Figure S8: Individual contribution. There are not significant differences in individual contribution by groups composition (ANOVA, F: 0.371 p: 0.9).

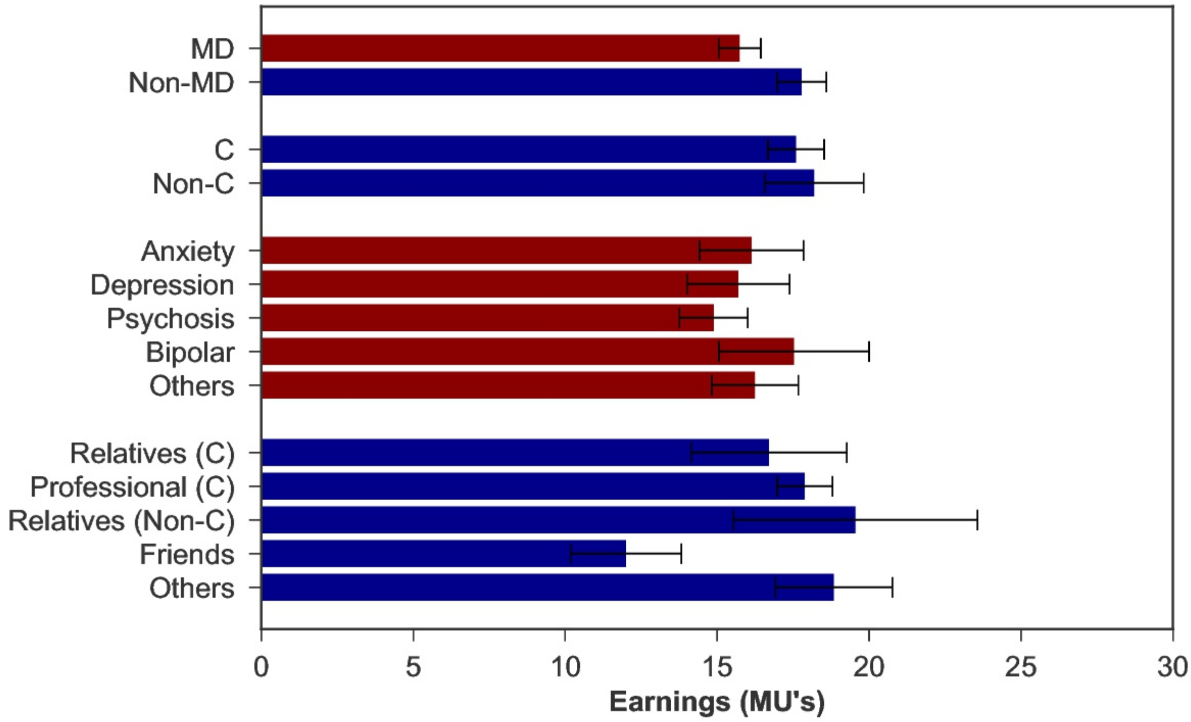

Supplementary Figure S9: Earnings in Colletive-Risk Social Dilemma. Earnings (mean and standard error of the mean) by role in the ecosystem. We show results from participants with and without Mental Disorder condition (MD and Non-MD, respectively), caregivers and non caregivers (C and Non-C, respectively), MD individuals with different diagnosis, and finally other actors that may and may not be caregivers.

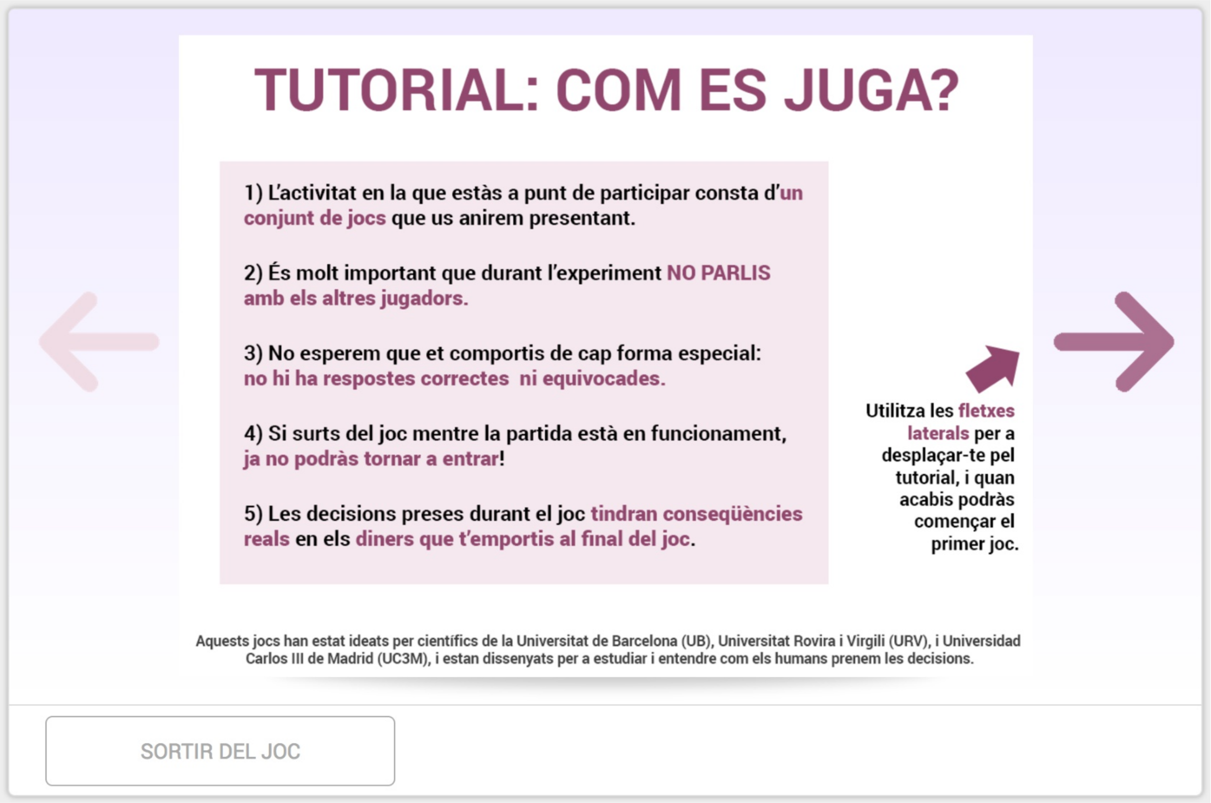

Supplementary Figure S10: Tutorial: How to play.

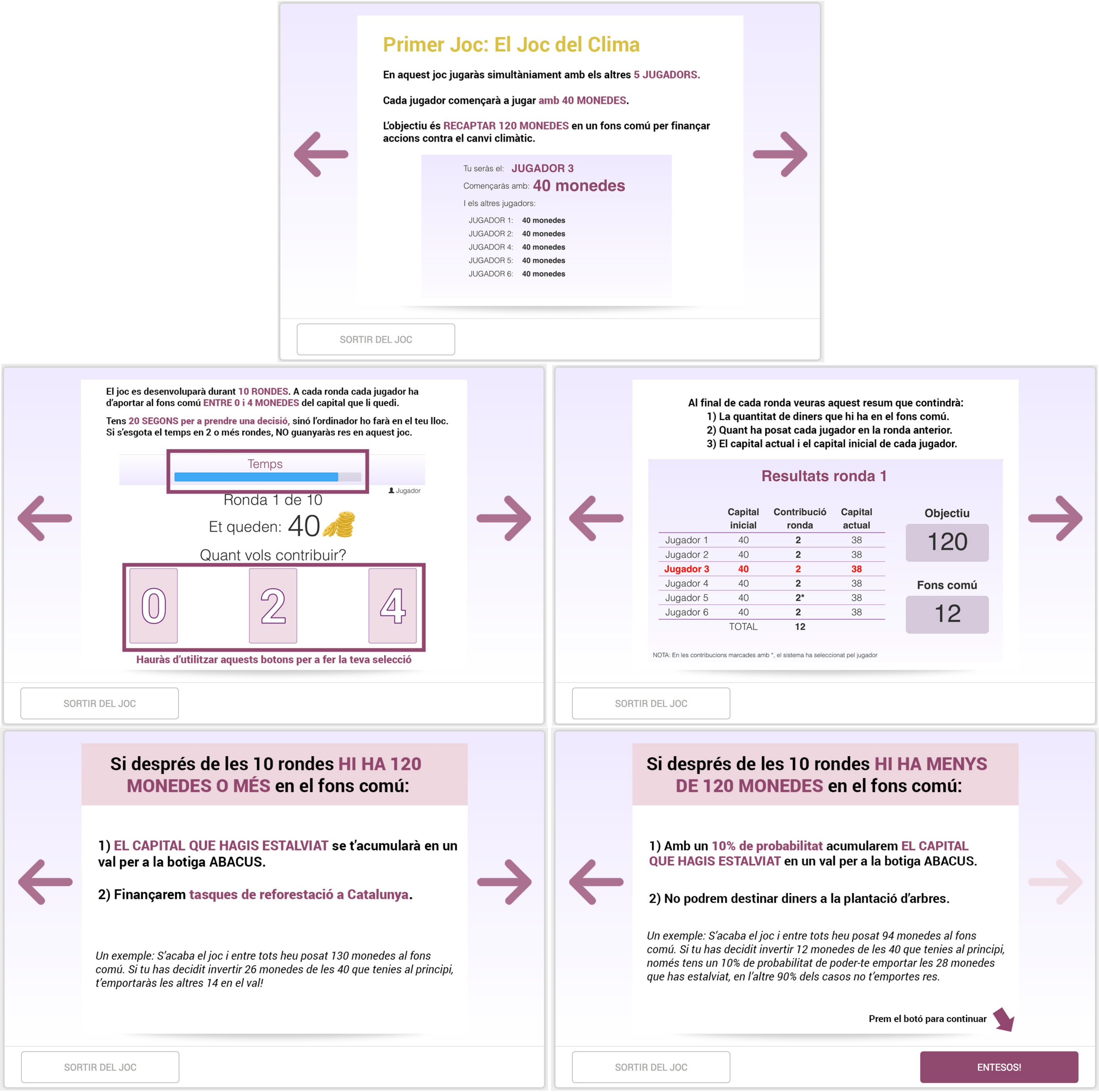

Supplementary Figure S11: Climate Game tutorial and game screenshots.

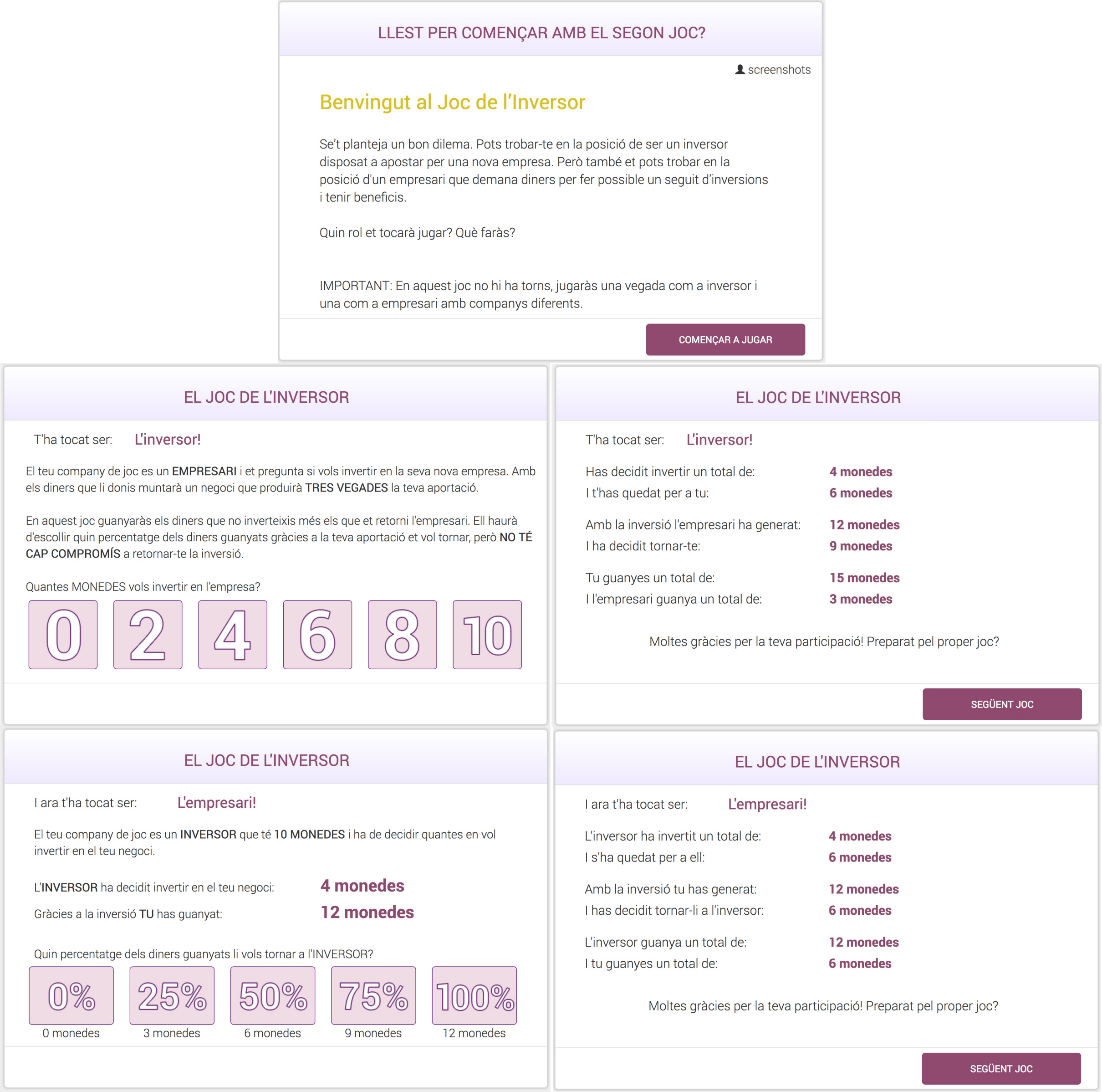

Supplementary Figure S12: Investor Game tutorial and game screenshots.

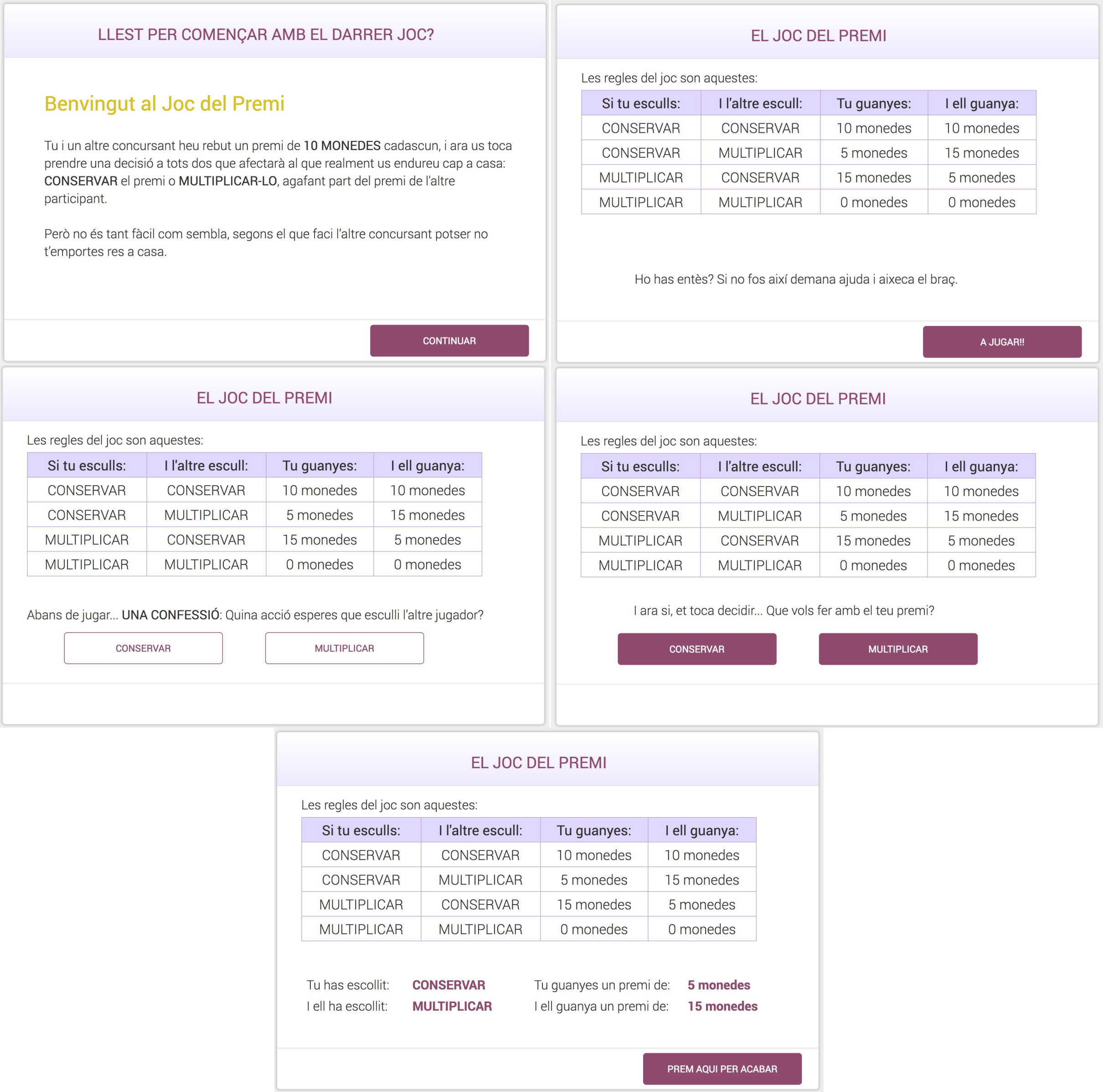

Supplementary Figure S13: Prize Game tutorial and game screenshots.

3 Supplementary Tables

Supplementary Table S1: Pairwise comparisons of cooperative behavior across diagnostics.

	**Bipolar**	**Pychosis**	**Depression**	**Anxiety**	**Others**
**Bipolar**		484 (0.48)	343.5 (0.13)	342 (0.045)**	261 (0.15)
**Pychosis**			1173 (0.23)	1173.5 (0.06)*	891.5 (0.26)
**Depression**				549 (0.53)	414 (0.97)
**Anxiety**					360.5 (0.58)
**Others**					

The table display the values of the Mann-Whitney-U tests (p-value) used to compare the frequency of cooperative decisions between each two diagnostics . *significant at 10%; **significant at 5%; ***significant at 1%.

Supplementary Table S2: Pairwise comparisons of expectations across diagnostics.

	**Bipolar**	**Pychosis**	**Depression**	**Anxiety**	**Others**
**Bipolar**		490 (0.54)	284.5 (0.92)	343.5 (0.03)**	231.5 (0.57)
**Pychosis**			966 (0.51)	1190 (0.04)**	791 (0.97)
**Depression**				659.5 (0.02)**	443.5 (0.57)
**Anxiety**					304.5 (0.09)*
**Others**					

The table display the values of the Mann-Whitney-U tests (p-value) used to compare the frequency of expected cooperation between each two diagnostics . *significant at 10%; **significant at 5%; ***significant at 1%.

Supplementary Table S3: Pairwise comparisons of trust across diagnostics.

	**Bipolar**	**Pychosis**	**Depression**	**Anxiety**	**Others**
**Bipolar**		466.5 (0.4)	311 (0.52)	297.5 (0.45)	217 (0.9)
**Pychosis**			1042.5 (0.98)	986.5 (0.93)	713.5 (0.48)
**Depression**				516.5 (0.94)	380 (0.6)
**Anxiety**					349.5 (0.52)
**Others**					

The table display the values of the Mann-Whitney-U tests (p-value) used to compare the initial transfers between each two diagnostics . *significant at 10%; **significant at 5%; ***significant at 1%.

Supplementary Table S4: Pairwise comparisons of reciprocity across diagnostics.

	**Bipolar**	**Pychosis**	**Depression**	**Anxiety**	**Others**
**Bipolar**		554 (0.81)	218 (0.18)	197.5 (0.13)	206 (0.86)
**Pychosis**			835.5 (0.097)*	761.5 (0.067)*	798 (0.92)
**Depression**				508.5 (0.97)	508 (0.11)
**Anxiety**					488 (0.07)*
**Others**					

The table display the values of the Mann-Whitney-U tests (p-value) used to compare the back transfers between each two diagnostics . *significant at 10%; **significant at 5%; ***significant at 1%.

Supplementary Table S5: Cooperation (c) and Expected Cooperation (c_{exp}) in Prisoner's Dilemma (mean±sem, standard error of the mean).

<table>
<tr><td colspan="10">Experimental individuals
$n = 270$</td></tr>
<tr><td colspan="10">$c = 0.61 \pm 0.03$
$c_{exp} = 0.59 \pm 0.03$</td></tr>
<tr><td colspan="5">Non-MD
$n = 101$</td><td colspan="5">MD
$n = 169$</td></tr>
<tr><td colspan="5">$c = 0.65 \pm 0.05$
$c_{exp} = 0.60 \pm 0.05$</td><td colspan="5">$c = 0.58 \pm 0.04$
$c_{exp} = 0.57 \pm 0.04$</td></tr>
<tr><td colspan="2">Caregivers
$n = 69$</td><td colspan="3">Non-Caregivers
$n = 32$</td><td>Anxiety
$n = 31$</td><td>Depression
$n = 33$</td><td>Psychosis
$n = 63$</td><td>Bipolar
$n = 17$</td><td>Others
$n = 25$</td></tr>
<tr><td colspan="2">$c = 0.72 \pm 0.05$
$c_{exp} = 0.65 \pm 0.06$</td><td colspan="3">$c = 0.5 \pm 0.09$
$c_{exp} = 0.5 \pm 0.09$</td><td>$c = 0.71 \pm 0.08$
$c_{exp} = 0.77 \pm 0.08$</td><td>$c = 0.64 \pm 0.09$
$c_{exp} = 0.48 \pm 0.09$</td><td>$c = 0.51 \pm 0.06$
$c_{exp} = 0.55 \pm 0.06$</td><td>$c = 0.41 \pm 0.12$
$c_{exp} = 0.47 \pm 0.12$</td><td>$c = 0.64 \pm 0.09$
$c_{exp} = 0.56 \pm 0.1$</td></tr>
<tr><td>Professional
$n = 52$</td><td>Relatives
$n = 17$</td><td>Relatives
$n = 9$</td><td>Friends
$n = 4$</td><td>Others
$n = 19$</td><td colspan="5"></td></tr>
<tr><td>$c = 0.67 \pm 0.07$
$c_{exp} = 0.63 \pm 0.07$</td><td>$c = 0.88 \pm 0.08$
$c_{exp} = 0.71 \pm 0.11$</td><td>$c = 0.33 \pm 0.16$
$c_{exp} = 0.44 \pm 0.18$</td><td>$c = 0.25 \pm 0.25$
$c_{exp} = 0.25 \pm 0.25$</td><td>$c = 0.63 \pm 0.11$
$c_{exp} = 0.59 \pm 0.12$</td><td colspan="5"></td></tr>
</table>

Supplementary Table S6: Trust (t) and Reciprocity (r) in Trust Game (mean±sem, standard error of the mean).

<table>
<tr><td colspan="10">Experimental individuals
$n = 270$</td></tr>
<tr><td colspan="10">$t = 0.58 \pm 0.01$
$r = 0.41 \pm 0.01$</td></tr>
<tr><td colspan="5">Non-MD
$n = 101$</td><td colspan="5">MD
$n = 169$</td></tr>
<tr><td colspan="5">$t = 0.55 \pm 0.02$
$r = 0.39 \pm 0.02$</td><td colspan="5">$t = 0.6 \pm 0.02$
$r = 0.43 \pm 0.02$</td></tr>
<tr><td colspan="2">Caregivers
$n = 69$</td><td colspan="3">Non-Caregivers
$n = 32$</td><td>Anxiety
$n = 31$</td><td>Depression
$n = 33$</td><td>Psychosis
$n = 63$</td><td>Bipolar
$n = 17$</td><td>Others
$n = 25$</td></tr>
<tr><td colspan="2">$t = 0.56 \pm 0.03$
$r = 0.41 \pm 0.02$</td><td colspan="3">$t = 0.54 \pm 0.04$
$r = 0.34 \pm 0.03$</td><td>$t = 0.61 \pm 0.05$
$r = 0.37 \pm 0.05$</td><td>$t = 0.6 \pm 0.05$
$r = 0.38 \pm 0.05$</td><td>$t = 0.61 \pm 0.03$
$r = 0.45 \pm 0.03$</td><td>$t = 0.56 \pm 0.06$
$r = 0.46 \pm 0.05$</td><td>$t = 0.57 \pm 0.06$
$r = 0.47 \pm 0.05$</td></tr>
<tr><td>Professional
$n = 52$</td><td>Relatives
$n = 17$</td><td>Relatives
$n = 9$</td><td>Friends
$n = 4$</td><td>Others
$n = 19$</td><td colspan="5"></td></tr>
<tr><td>$t = 0.57 \pm 0.03$
$r = 0.42 \pm 0.03$</td><td>$t = 0.51 \pm 0.05$
$r = 0.4 \pm 0.03$</td><td>$t = 0.46 \pm 0.06$
$r = 0.44 \pm 0.07$</td><td>$t = 0.7 \pm 0.06$
$r = 0.25 \pm 0.1$</td><td>$t = 0.54 \pm 0.07$
$r = 0.32 \pm 0.04$</td><td colspan="5"></td></tr>
</table>
